# Prognostic assessment of the Japanese Renal Pathology Society classification in Chinese patients with histologically confirmed diabetic kidney disease

**DOI:** 10.1007/s10157-025-02782-w

**Published:** 2025-11-26

**Authors:** Ying Shi, Yuyou Ye, Qian Zhou, Hujia Hua, Yanggang Yuan, Chengning Zhang, Huijuan Mao, Suyan Duan, Bo Zhang

**Affiliations:** 1 The First Affiliated Hospital with Nanjing Medical University, Jiangsu Province Hospital, Nanjing Medical University, Nanjing, People’s Republic of China; 2 Xi’an No.3 Hospital, the Affiliated Hospital of Northwest University, Xi’ an, Shaanxi People’s Republic of China

**Keywords:** Diabetic kidney disease, Kidney biopsy, JRPS classification, Renal outcomes

## Abstract

**Background:**

This study aimed to comparatively evaluate the prognostic value of the Japanese Renal Pathology Society (JRPS) classification for predicting diabetic kidney disease (DKD) progression in Chinese patients.

**Methods:**

This retrospective cohort study included 124 patients diagnosed with DKD from 2014 to 2020. Patients were classified into four JRPS classification grades based on the J-score. Renal survival was assessed using Kaplan–Meier analysis and Cox regression, and predictive accuracy was compared with the RPS classification and total renal chronicity score using receiver operating characteristic (ROC) curve analysis and the DeLong test.

**Results:**

Over a median follow-up of 37 months, 76.6% of patients reached renal outcomes, including 40.3% progressing to end-stage kidney disease (ESKD). Higher JRPS classification grades were independently associated with adverse renal outcomes. However, ROC analysis demonstrated that the JRPS classification exhibited inferior discriminative performance compared with the traditional RPS classification system.

**Conclusion:**

The JRPS classification was independently associated with renal outcomes but showed inferior discriminatory performance compared with the RPS classification. These findings suggest that JRPS classification may provide complementary pathological information rather than serving as a primary prognostic tool.

**Supplementary Information:**

The online version contains supplementary material available at 10.1007/s10157-025-02782-w.

## Introduction

The global prevalence of diabetes is rising, with over 500 million affected individuals in 2023, and 30–40% of them are expected to develop diabetic kidney disease (DKD) [1, 2]. DKD is a leading cause of end-stage kidney disease (ESKD) and is strongly associated with cardiovascular morbidity and mortality [3].

Pathological classification is crucial for prognosis assessment in DKD. The Renal Pathology Society (RPS) classification, initially introduced by Tervaert et al. [4] in 2010, has been widely used to predict renal prognosis in DKD. However, this classification primarily focuses on glomerular changes in the kidney, while numerous studies suggest that tubulointerstitial changes also play a significant role in the progression of DKD [5]. In 2017, Sethi et al. proposed the "total renal chronicity score" system, which evaluates glomerulosclerosis, tubular atrophy, interstitial fibrosis, and arteriosclerosis using semi-quantitative scoring to calculate a total chronic disease grade [6]. However, its evaluation of glomerular lesions remains relatively simplistic.

In 2018, the Japanese Renal Pathology Society (JRPS) proposed the JRPS score (J-score), which is a comprehensive DKD classification system [7]. The J-score encompasses various pathological findings, such as glomerular, tubulointerstitial, and vascular lesions. Nevertheless, only a few studies have been conducted to assess the clinical usefulness of the JRPS classification.

This study aims to evaluate the prognostic value of the JRPS classification in a Chinese DKD cohort, assess its association with renal disease progression, and compare its prognostic performance with other pathological classification systems.

## Materials and methods

### Subjects

We retrospectively enrolled 124 patients with biopsy-proven DKD between January 2014 and September 2020 at the Department of Nephrology, First Affiliated Hospital with Nanjing Medical University [8]. The study was approved by the Ethics Committee (Approval No. 2024-SR-297) and was conducted in accordance with the 1964 Helsinki Declaration and its subsequent amendments or comparable ethical standards. All enrolled patients provided informed consent at the time of their renal biopsy. (Supplementary Fig. S1).

### Clinical and pathological parameters

The complete clinical and laboratory data of enrolled patients at the time of renal biopsy were collected from electronic medical records, including age, sex, clinical symptoms, past medical history, and laboratory test results. Additionally, we recorded the medication history, blood pressure-lowering therapy (renin–angiotensin–aldosterone system inhibitors (RAASi) and calcium-channel blocker (CCB)), statins, insulin, and antihyperglycemic agents. The estimated glomerular filtration rate (eGFR) was calculated using the Chronic Kidney Disease Epidemiology Collaboration (CKD-EPI) equation.

Histopathological diagnoses were made by two experienced renal pathologists, who were blinded to the patients' clinical information. Scoring discrepancies were resolved by consensus. Kidney biopsy specimens were examined using light microscopy, immunofluorescence, and electron microscopy, following standard protocols. The renal specimens were assessed using the RPS glomerular classification, which includes key features such as glomerular basement membrane (GBM) thickening, mesangial expansion, nodular sclerosis, and global sclerosis [4]. Additionally, the revised classification by the Japanese Renal Pathology Society (JRPS) was utilized, which includes diffuse lesions, nodular lesions, widening of the subendothelial space, mesangiolysis, exudative lesions, perihilar neovascularization, global and segmental glomerulosclerosis, glomerulomegaly, interstitial fibrosis and tubular atrophy (IFTA), interstitial inflammation, arteriolar hyalinosis (AH), and intimal thickening. The J-score, as described by Hoshino et al., was used to categorize patients into four groups: JRPS classification grade 1 (J-score 0–5), grade 2 (J-score 6–10), grade 3 (J-score 11–15), and grade 4 (J-score 16–19) [7].

Furthermore, renal chronicity scoring and grading followed the criteria outlined by Sethi et al. [6]. Glomerulosclerosis, tubular atrophy, and interstitial fibrosis were scored on a scale from 0 to 3 based on the percentage of tissue involvement: 0 (< 10%), 1 (10–25%), 2 (26–50%), and 3 (> 50%). Arteriosclerosis was scored as 0 if the intimal thickness was less than the medial thickness, and as 1 if the intimal thickness exceeded the medial thickness. The individual scores were then summed, and chronicity was graded as follows: 0–1 (minimal), 2–4 (mild), 5–7 (moderate), and ≥ 8 (severe).

### Study outcomes

The study outcomes were divided into renal and cardiovascular outcomes. The renal outcomes was a composite of a sustained doubling of serum creatinine (D-Scr) or progression to end-stage kidney disease (ESKD; dialysis, transplantation, or eGFR < 15 mL/min/1.73 m^2^). The cardiovascular outcomes included major events such as myocardial infarction or revascularization, stroke, peripheral vascular disease, and cardiovascular death. [3].

### Statistical analysis

Continuous variables were compared using ANOVA or the Kruskal–Wallis test, and categorical variables using the χ^2^ or Fisher’s exact test. Renal survival was evaluated by Kaplan–Meier and log-rank tests. Associations between pathological classifications and renal outcomes were analyzed with Cox regression, adjusted for key demographic and clinical factors. Model discrimination was assessed by receiver operating characteristic (ROC) and DeLong test, and clinical utility by decision curve analysis (DCA). Calibration and fit were evaluated using the − 2 log-likelihood ratio and Hosmer–Lemeshow χ^2^ test. Reclassification was quantified by net reclassification improvement (NRI) and integrated discrimination improvement (IDI). Analyses were performed with SPSS 27.0 and R 4.4.2; two-sided *P* < 0.05 was considered significant.

## Results

### Clinical and pathological baseline characteristics across JRPS classification grades of DKD

A total of 124 patients with DKD were enrolled, with a mean age of 50.27 ± 11.32 years. Among them, 97 (78.23%) were male. We further analyzed the clinicopathological characteristics of the patients, stratified by the JRPS classification grades of DKD. Over a median follow-up period of 40.58 ± 23.02 months, 95 (76.6%) experienced composite renal outcomes, including 50 (52.63%) with ESKD and 45 (47.3%) with D-Scr. The incidence of renal outcomes increased progressively with higher JRPS classification grades (*P* < 0.05). Although patients in higher JRPS classification grades had a numerically higher rate of cardiovascular events, the difference was not statistically significant (*P* = 0.492).

Higher grades were associated with higher levels of serum creatinine, blood urea nitrogen (BUN), and cystatin C, while lower grades were associated with higher levels of hemoglobin, high-density lipoprotein cholesterol (HDL-C), and eGFR. Other clinical parameters did not differ significantly among the grades (Table 1).

**Table 1:** Baseline clinical and laboratory data for all enrolled patients according to JRPS classification grades

	Grade 1 (*n* = 14)	Grade 2 (*n* = 26)	Grade 3 (*n* = 60)	Grade 4 (*n* = 24)	*P*
Age (years)	52.93 ± 13.43	52.12 ± 11.07	49.18 ± 12.47	49.25 ± 7.97	0.545
Gender (male %)	23.70	41.20	35.10	35.10	0.117
Follow-up time (months)	52.00 (40.75,71.97)	37.50 (34.85,54.69)	35.50 (31.26,41.91)	32.00 (26.35,43.76)	0.055
Body mass index (kg/m2)	26.05(23.37,28.73)	26.32(24.73,27.91)	24.64(23.91,25.37)	26.50(24.67,28.32)	0.313
Duration of diabetes (year)	11.07(7.92,14.22)	10.17(6.99,13.35)	9.69(7.92,11.46)	9.72(6.80,12.63)	0.120
SBP (mmHg)	147.57(133.31,161.83)	155.54(143.95,167.13)	148.79(142.21,155.37)	142.83(135.35,150.32)	0.266
DBP (mmHg)	86.93(78.80,95.06)	91.19(84.02,98.37)	87.56(83.62,91.50)	83.67(79.06,88.27)	0.610
Cardiovascular outcomes (%)	6(42.85)	7(26.92)	20(76.92)	11(45.83)	0.492
Renal outcomes (%)	9(64.29)	16(61.54)	46(76.67)	24(100.00)	< 0.001
D-Scr (%)	6(42.86)	5(19.20)	22(36.67)	12(50.00)	0.139
ESKD (%)	3(21.43)	11(42.31)	25(41.67)	11(45.83)	0.481
Hemoglobin(g/L)	128.00(111.33,137.38)	117.00(09.65,126.58)	105.50(102.89,114.24)	107.00(100.75,114.42)	< 0.05
HbA1c(%)	7.55(7.56,8.92)	7.50(7.13,8.81)	7.10(7.20,8.27)	6.8(6.57,7.60)	0.266
FBG (mmol/L)	8.54(6.81,11.22)	6.32(6.02,8.07)	6.10(6.44,9.70)	7.57(6.71,10.65)	0.272
Scr (μmol/L)	90.85(77.72,106.81)	103.95(94.51,170.61)	108.00(119.34,162.83)	183.25(153.12,223.94)	< 0.05
BUN (mmol/L)	6.90(5.96,8.05)	8.93(7.20,12.23)	8.73(8.49,11.15)	10.54(9.56,14.81)	< 0.05
Uric acid(mmol/L)	318.00(302.53,371.64)	378.00(351.48,422.60)	372.20(355.47,401.44)	397.50(373.60,448.56)	0.096
Serum albumin (g/L)	36.50(28.13,37.80)	31.00(28.60,34.35)	30.65(30.17,34.4)	32.90(30.71,35.73)	0.372
Total cholesterol (mmol/L)	5.21(4.66,6.08)	4.80(4.22,5.70)	5.54(5.35,6.14)	5.23(4.50,5.71)	0.132
Triglyceride (mmol/L)	1.38(1.13,2.57)	1.47(1.24,2.37)	1.51(1,71,2.87)	1.85(1.64,3.19)	0.254
HDL-C (mmol/L)	1.15(1.08,1.34)	1.00(0.93,1.19)	1.14(1.13,1.34)	0.97(0.86,1.04)	< 0.05
LDL-C (mmol/L)	3.28(2.94,4.00)	3.12(2.68,3.70)	3.54(3.38,4.00)	3.05(2.93,3.84)	0.296
eGFR (ml/min/1.73 m^2^)	74.97(67.30,90.18)	70.62(54.24,81.66)	65.57(55.32,72.04)	32.67(30.94,50.85)	< 0.05
Urinary protein (g/d)	2.69(1.30,6.11)	3.63(2.99,5.98)	3.59(4.11,6.43)	4.04(3.21,9.78)	0.274
Cystatin C(mg/L)	1.42(1.3,1.55)	1.62(1.47,1.76)	1.99(1.76,2.21)	1.99(1.76,2.21)	< 0.05
PTH (pg/ml)	49.05(32.03,54.48)	45.50(42.60,72.67)	52.40(49.68,71.60)	63.00(49.09,135.04)	0.562
Ca(mmol/L)	2.13(1.91,2.27)	2.14(2.08,2.20)	2.11(2.07,2.16)	2.14(2.11,2.20)	0.606
P(mmol/L)	1.24(1.05,1.32)	1.24(1.16,1.30)	1.27(1.23,1.33)	1.27(1.22,1.36)	0.421
IgA(g/L)	2.67(2.09,3.18)	2.35(2.25,3.40)	2.45(2.32,2.83)	2.02(1.91,2.46)	0.26
IgG(g/L)	11.26 ± 4.80	10.61 ± 3.28	9.87 ± 3.29	9.10 ± 2.25	0.199
C3(g/L)	1.09 ± 0.21	1.11 ± 0.21	1.04 ± 0.18	1.12 ± 0.15	0.265
C4(g/L)	0.29(0.21,0.32)	0.28(0.26,0.33)	0.28(0.26,0.30)	0.29(0.27,0.32)	0.723
RAASi (%)	13(92.86)	24(92.31)	47(78.33)	19(79.17)	0.239
CCB (%)	9(64.29)	18(69.23)	40(66.67)	16(66.67)	0.991
		14(53.85)	33(55.00)	14(58.33)	
Antihyperglycemic agents (%)	10(71.43)	14(53.85)	33(55.00)	14(58.33)	0.703
Insulin (%)	11(78.57)	19(73.08)	51(85.00)	18(75.00)	0.552
Statin (%)	3(21.43)	12(46.15)	32(53.33)	9(37.50)	0.124

*SBP* Systolic blood pressure, *DBP* Diastolic blood pressure, *eGFR* Estimated glomerular filtration rate, *BUN* Blood urea nitrogen, *Scr* Serum creatinine, *FBG* Fasting blood glucose, *LDL-C* low-density lipoprotein cholesterol, *HDL-C* High-density lipoprotein cholesterol, *PTH* Parathyroid hormone, *RAASi* Renin–angiotensin–aldosterone system inhibitors, *CCB* Calcium-channel blocker, *D-Scr* Doubling of serum creatinine level, *ESKD* End stage kidney disease

Data were presented as the mean ± standard, the median with interquartile range or counts and percentages

A two-tailed *P* < 0.05 was considered statistically significant

The baseline pathological features also differed significantly across JRPS classification (Table 2). Higher JRPS classification grades were associated with markedly greater pathological severity. The total renal chronicity score and J-score increased progressively with grade, as did the extent of diffuse and nodular lesions, subendothelial space widening, exudative lesions, perihilar neovascularization, IFTA, interstitial cell infiltration, arteriolar hyalinosis, and global glomerulosclerosis.

**Table 2:** Baseline pathological characteristics of patients

Parameter	Grade 1(*n* = 14)	Grade 2 (*n* = 26)	Grade 3 (*n* = 60)	Grade 4 (*n* = 24)	*P*
RPS glomerular class	2/7/5/0	3/10/12/1	0/14/32/14	0/1/11/12	< 0.001
Total renal chronicity score	1.43 ± 1.09	2.35 ± 1.79	3.78 ± 2.73	5.13 ± 1.70	< 0.001
Total renal chronicity grade	8/6/0/0	10/13/3/0	15/21/18/6	0/9/13/2	< 0.001
J-score	3.29 ± 1.20	8.54 ± 0.86	12.78 ± 1.50	16.75 ± 0.85	< 0.001
Diffuse(1/2/3)	7/7/0	18/8/0	24/29/7	5/12/7	< 0.001
Nodular(0/1)	9/5	14/32	20/40	6/18	< 0.05
SubendW(0/1/2/3)	0/8/6/0	0/12/13/1	0/16/29/15	0/1/7/16/24	< 0.001
Exudative (0/1)	14/0	22/4	37/23	0/24	< 0.001
MesLy (0/1)	6/8	13/13	20/40	8/16	0.474
PVas (0/1)	10/4	20/6	26/34	5/19	< 0.001
GScl (%)	15.08(9.16,2.75)	27.20(18.92,37.90)	21.92(22.32,35.20)	42.58(31.46,55.47)	< 0.05
SScl (%)	11.25(6.86,28.67)	17.39(14.99,30.33)	19.15(14.85,23.95)	10.82(7.91,19.10)	0.305
GMeg (0/1)	5/9	8/18	23/37	9/15	0.926
IFTA (0/1/2/3)	4/10/0/0	0/16/5/5	1/11/20/28	0/2/7/15	< 0.001
ICell (0/1/2)	2/1/11	1/16/9	0/35/25	0/9/15/24	< 0.001
Hyalin (0/1/2/3)	8/6/0/0	22/3/0/1	20/26/0/14	5/17/0/2	< 0.001
IntThic (0/1/2)	1/12/1	1/23/2	0/54/6	0/18/6	0.188

*JRPS* Japanese renal pathology society, *J-score* JRPS score, *RPS* Renal pathology society, *Diffuse* Diffuse lesion (mesangial expansion), *Nodular* Nodular lesion (nodular sclerosis), *SubendW* Subendothelial space widening (double contour of basement membrane), *Exudative* Exudative lesion, *MesLy* Mesangiolysis/microaneurysm, *PVas* Perihilar neovascularization (polar vasculosis), *GScl* Global glomerulosclerosis/collapsing glomerular change ischemic glomerular change, *SScl* Segmental glomerulosclerosis, *GMeg* Glomerulomegaly, *IFTA* Interstitial fibrosis and tubular atrophy, *ICell* Interstitial cell infiltration, *Hyalin* Arteriolar hyalinosis *Arterio* Arteriosclerosis with intimal thickening

### Correlations between JRPS classification pathological factors and clinical factors

**Fig. 1:**
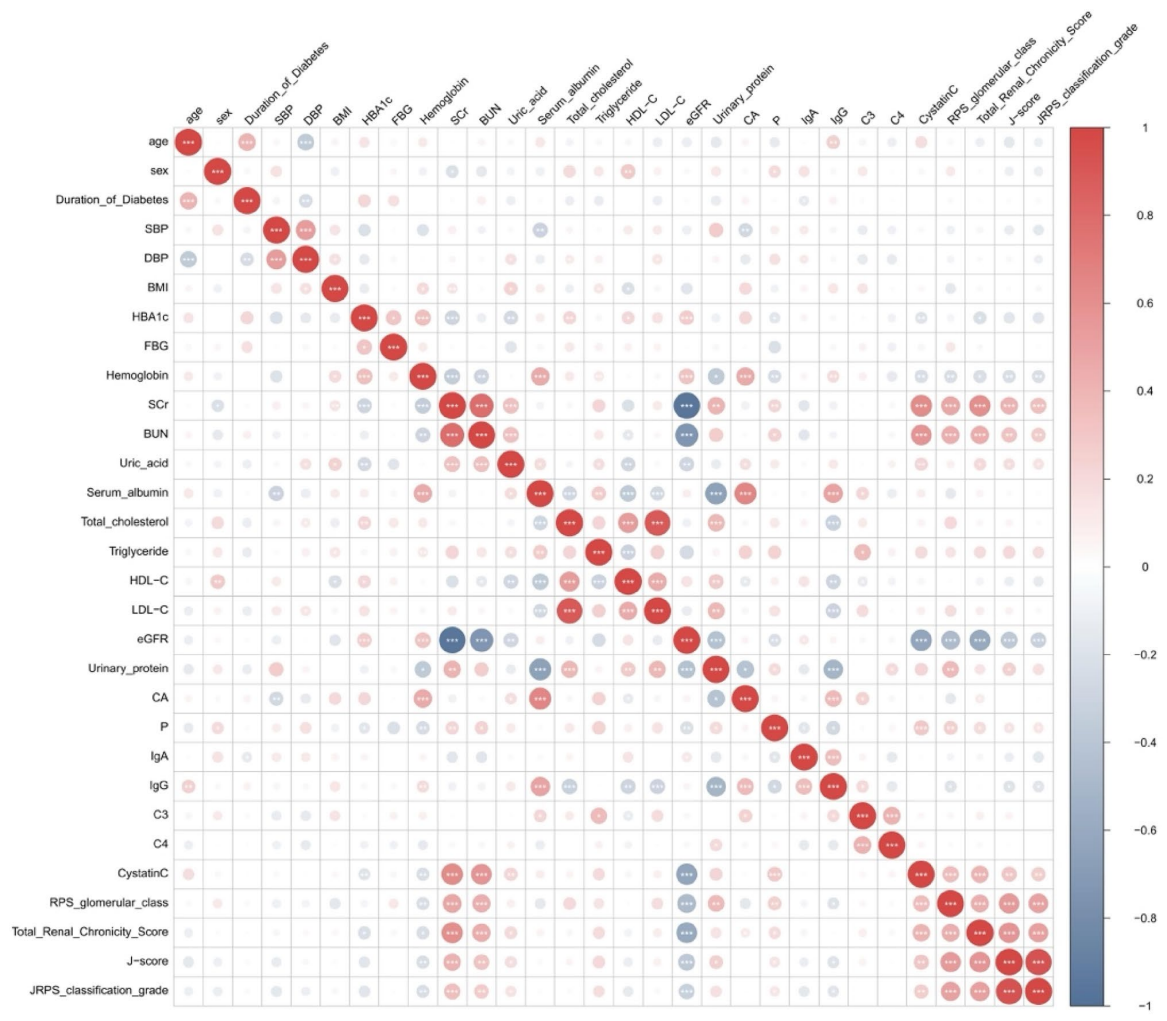
Correlations Heatmap of JRPS classification with clinicopathological characteristics. *SBP* Systolic blood pressure, *DBP* Diastolic blood pressure, *eGFR* Estimated glomerular filtration rate, *BUN* Blood urea nitrogen, *Scr* Serum creatinine, *FBG* Fasting blood glucose, *LDL-C* Low-density lipoprotein cholesterol, *HDL-C* High-density lipoprotein cholesterol, *JRPS* Japanese renal pathology society, *J-score* JRPS score, *RPS* Renal pathology society

Correlation analysis showed that the J-score was negatively correlated with eGFR and hemoglobin, and positively correlated with serum creatinine (Scr), BUN, uric acid (UA), cystatin C, urinary protein, and triglycerides (TG). Similarly, the overall JRPS pathological grade was significantly negatively correlated with eGFR and hemoglobin, but positively correlated with Scr, BUN, and cystatin C (Fig. 1). Specific lesions including nodular sclerosis, mesangiolysis, glomerulosclerosis, and IFTA were also associated with lower albumin and higher proteinuria (Table 3). To further illustrate these relationships, Supplementary Figure S2 presents representative boxplots and strip plots with overlaid individual data points showing the associations between key pathological parameters and clinical variables.

**Table 3:** Correlations between JRPS classification pathological factors and clinical factors

Parameter	Correlation coefficient (r)					
	Hemoglobin	Scr	BUN	Serum albumin	eGFR	Urinary protein
Diffuse(0/1/2/3)	− 0.16	0.23*	0.15	− 0.11	− 0.16	0.11
Nodular(0/1)	− 0.34***	0.13	0.16	− 0.42***	− 0.19*	0.44****
SubendW(0/1/2/3)	− 0.27**	0.39****	0.42***	− 0.17	0.52****	0.38****
Exudative (0/1)	− 0.14	0.24**	0.2*	− 0.07	− 0.27**	0.21*
MesLy (0/1)	− 0.23*	0.13	0.06	− 0.28**	− 0.21*	0.26**
PVas (0/1)	− 0.23**	0.2	0.27**	− 0.12	− 0.28**	0.33***
GScl (%)	0.01	0.4****	0.28**	0.24**	− 0.47***	−0.03
SScl (%)	− 0.16	− 0.07	0	− 0.23*	0.08	0.15
GMeg (0/1)	− 0.15	0.05	0.01	− 0.2*	0	0.26**
IFTA (0/1/2/3)	− 0.3***	0.38****	0.25**	− 0.08	− 0.41***	0.17
ICell (0/1/2)	− 0.23**	0.28**	0.17	− 0.12	− 0.33***	0.15
Hyalin (0/1/2/3)	− 0.03	0.02	0	− 0.02	− 0.06	0.11
IntThic (0/1/2)	0.05	0.13	0.14	0.18*	− 0.17	0.01

*Diffuse* Diffuse lesion (mesangial expansion), *Nodular* Nodular lesion (nodular sclerosis), *SubendW* Subendothelial space widening (double contour of basement membrane), *Exudative* Exudative lesion, *MesLy* Mesangiolysis/microaneurysm, *PVas* perihilar neovascularization (polar vasculosis), *GScl* Global glomerulosclerosis/collapsing glomerular change ischemic glomerular change, *SScl* Segmental glomerulosclerosis, *GMeg* Glomerulomegaly, *IFTA* Interstitial fibrosis and tubular atrophy, *ICell* Interstitial cell infiltration, *Hyalin* Arteriolar hyalinosis, *Arterio* Arteriosclerosis with intimal thickening, *eGFR* Estimated glomerular filtration rate

^*^P < 0.05, **P < 0.01, ***P < 0.001, ****P < 0.0001

### Correlations of JRPS classification with RPS glomerular class and the total renal chronicity grade

**Fig. 2:**
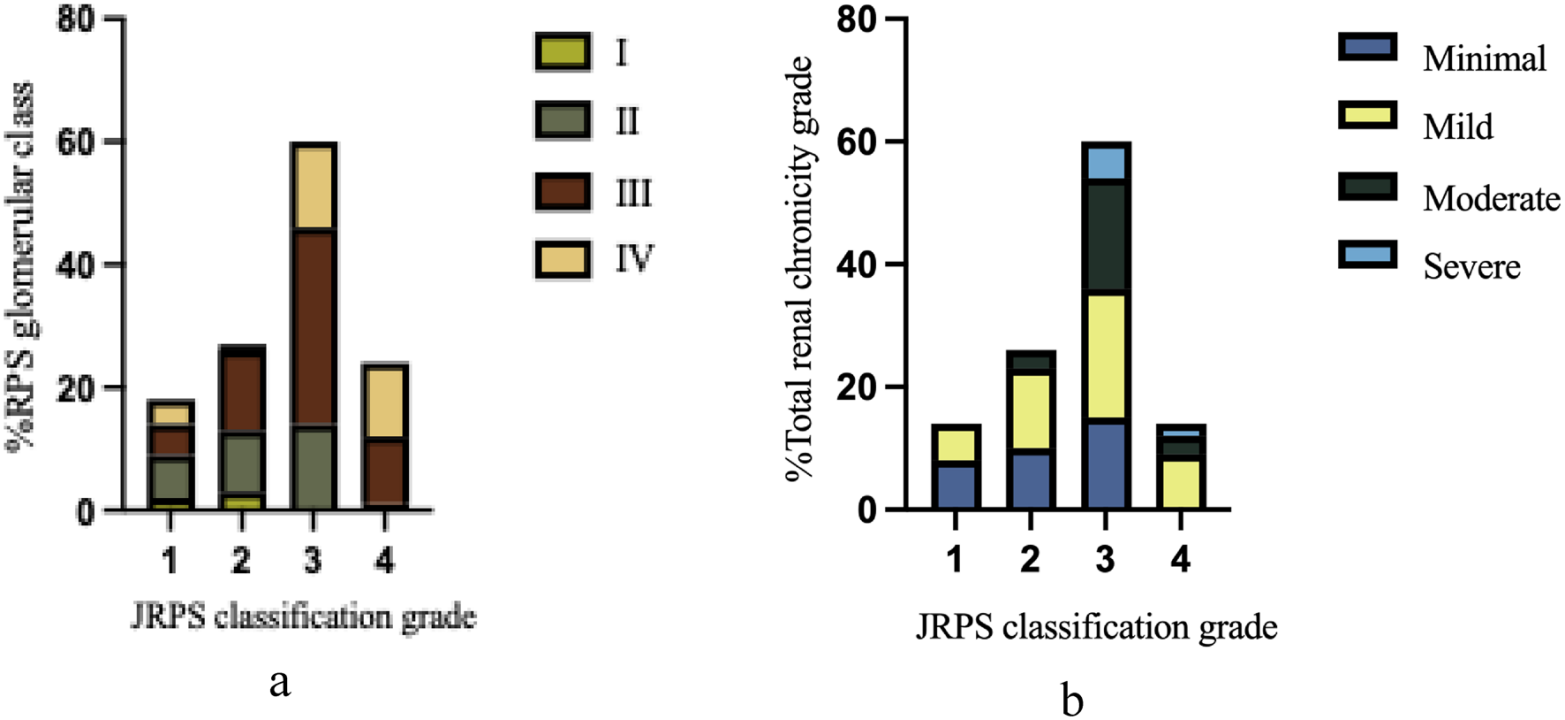
The percentage for RPS glomerular class (**a**) and total renal chronicity grade based (**b**) on the JRPS classification grade. *JRPS* Japanese renal pathology society, *J-score* JRPS score, *RPS* Renal pathology society

Most patients classified as RPS glomerular class I or II were distributed within the lower JRPS classification grades, whereas those with RPS class IV were predominantly concentrated in the higher JRPS classification grades. The proportion of patients with RPS class III increased progressively with higher JRPS classification grades, representing 4.0%, 9.7%, and 25.8% in JRPS classification grades 1, 2, and 3, respectively (Fig. 2a).

Similarly, patients with minimal chronicity were mainly clustered within the lower JRPS classification grades (1–3), while those with moderate or severe chronicity were more commonly observed in the higher JRPS classification grades (3–4). Patients with mild chronicity were more evenly distributed across both lower and higher JRPS classification grades. (Fig. 2b).

### Associations of Japanese renal pathology society classification with renal outcomes

**Fig. 3:**
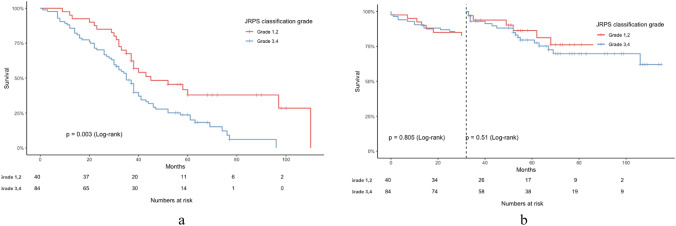
Kaplan–Meier survival curves stratified by JRPS classification grades (1–2 vs. 3–4). **A** Renal outcome-free survival in patients stratified by JRPS grades (grouped as 1–2 vs 3–4). Log-rank test was used to assess significance. (*P* < 0.05 considered significant). **B** Landmark analysis of cardiovascular event-free survival at 32 months. No significant difference was observed between JRPS risk groups during follow-up

During a median follow-up of 37.00 (36.15, 44.32) months, 95 patients (76.6%) experienced the composite renal outcomes, including 50 patients (52.63%) with ESKD and 45 patients (47.37%) with D-Scr. Additionally, 44 patients (35.5%) experienced cardiovascular outcomes. Kaplan–Meier survival analysis showed that the cumulative incidence of primary renal outcomes increased significantly with higher JRPS classification grades (log-rank *P* = 0.003), indicating that patients with higher JRPS classification grades had significantly worse renal outcomes (Fig. 3a). Landmark analysis, for the cardiovascular outcomes, using 32 months as the landmark time, demonstrated that JRPS classification grade was not a significant predictor of cardiovascular survival in the early stage, however, its prognostic effect became more evident after 32 months. (Fig. 3b).

Univariate Cox analysis identified that patients with higher degrees of diffuse lesions, nodular lesions, subendothelial space widening, mesangiolysis, exudative lesions, perihilar neovascularization, glomerulomegaly, and more advanced IFTA were at a higher risk of renal function deterioration. These renal pathological changes were significant predictors of the occurrence of composite kidney events (shown in Supplementary Table 1).

Multivariate Cox regression confirmed that higher JRPS classification grades were independently associated with adverse renal outcomes. In unadjusted models, patients with higher JRPS classification grades were associated with higher risks of renal outcome occurrence. These associations remained significant after adjustment for age and gender in model 2. Furthermore, after adjustment for age, gender, urinary protein, Scr, duration of diabetes, use of RAASi, CCB, higher JRPS classification grades still had significantly increased risk for renal outcomes. (JRPS classification grade 3, 4 HR:1.74 [1.07–2.85], *P* = 0.026; model 3 shown in Table 4) Consistent findings were observed in additional multivariable Cox models for the J-score, RPS glomerular class, and total chronicity grade (Supplementary Tables S2–S4).

**Table 4:** Effect of JRPS classification grade on renal outcomes

Variables	Model 1			Model 2			Model 3	
	HR (95% CI)	*P*		HR (95% CI)	*P*		HR (95% CI)	*P*
JRPS classification grade								
Grade 1, 2	1.00 (Reference)			1.00 (Reference)			1.00 (Reference)	
Grade 3, 4	2.02 (1.25–3.25)	0.004		1.97 (1.22–3.20)	0.006		1.74 (1.07–2.85)	0.026

Model 1: Crude

Model 2: Adjust: age, gender

Model 3: Adjust: age, gender, urinary protein, duration of diabetes, Scr, use of RAASi, statins, CCB

*HR* Hazard ratio, *CI* Confidence interval, *JRPS* Japanese renal pathology society, *Scr* Serum creatinine, *RAASi* Renin–angiotensin–aldosterone system inhibitors, *CCB* Calcium-channel blocker

Subgroup analysis further confirmed the association between the pathological grades of JRPS and the risk of renal progression. Higher pathological grades of JRPS were associated with an increased risk of renal progression (JRPS classification grade 3: HR 2.37 [1.09–5.17], *P* = 0.03; JRPS classification grade 4: HR 3.36 [1.47–7.66], *P* = 0.03). Although the interaction terms were not statistically significant, higher risk estimates were observed among subgroups including older patients (age > 55 years), males, individuals with advanced CKD, those with SBP ≤ 140 mmHg, BMI > 25 kg/m^2^, and patients receiving RAAS inhibitors, antihyperglycemic agents, or insulin (Table 5).

**Table 5:** Subgroup analyses of the association between JRPS classification grade and renal outcomes in patients with DKD

Characteristic	HR (95% CI), P-value				P for interaction
	Grade 1	Grade 2	Grade 3	Grade 4	
All patients	1 (reference),1	1.5(0.64,3.56),0.353	2.37(1.09,5.17),0.03	3.36(1.47,7.66),0.004	
Age					0.54
≤ 55 years	1 (reference),1	0.92(0.28,3.04),0.896	1.99(0.7,8.01),0.195	2.51(0.85,7.4),0.095	
> 55 years	1 (reference),1	2.61(0.75,9.12),0.133	2.48(0.77,8.01),0.129	3.87(1,14.95),0.05	
Gender					0.434
Female	1 (reference),1	1.28(0.25,6.65),0.768	0.99(0.21,4.56),0.989	1.06(0.14,7.81),0.955	
Male	1 (reference),1	1.5(0.54,4.13),0.432	2.8(1.12,6.79),0.027	4.04(1.58,10.35),0.004	
Serum albumin					0.303
≤ 30 g/L	1 (reference),1	2.47(0.69,8.92),0.167	2.43(0.73,8.07),0.147	5.57(1.43,21.63),0.013	
> 30 g/L	1 (reference),1	0.85(0.24,3.01),0.797	2.36(0.83,6.74),0.109	3.95(1.35,11.53),0.012	
CKD Stages					0.584
Stages 1–2	1 (reference),1	1.25(0.27,5.83),0.733	1.41(0.33,5.97),0.64	1.52(0.35,6.62)0.58	
Stages 3–4	1 (reference),1	1.12(0.37,3.37),0.844	2(0.77,5.17),0.153	3.35(1.04,10.74,0.042)	
SBP					0.369
≤ 140 mmHg	1 (reference),1	4.87(0.55,43.04)),0.155	7.83(1.02,60.3),0.048	11.02(1.43,84.75),0.021	
> 140 mmHg	1 (reference),1	1.01(0.39,2.61),0.991	1.5(0.65,3.48),0.346	2.22(0.81,6.06),0.12	
BMI					0.852
≤ 25 kg/m2	1 (reference),1	1.36(0.44,4.16),0.592	1.79(0.68,4.72),0.236	2.67(0.9,7.97),0.078	
> 25 kg/m2	1 (reference),1	1.69(0.44,6.53),0.444	2.99(0.85,10.47),0.088	3.99(1.13,14.06),0.031	
Proteinuria					0.335
≤ 3.5 g/d	1 (reference),1	1.18(0.31,4.52),0.813	2.67(0.84,8.48),0.095	5.15(1.5,17.75),0.009	
> 3.5 g/d	1 (reference),1	1.48(0.47,4.64),0.506	1.62(0.57,4.65),0.369	1.85(0.59,5.75),0.289	
Use of RAASi					0.198
NO	1 (reference),1	0.26(0.02,4.39),0.349	0.81(0.1,6.62),0.841	1.98(0.22,17.69),0.542	
YES	1 (reference),1	1.83(0.73,4.54),0.195	2.58(1.11,5.98),0.027	3.32(1.36,8.09),0.008	
Use of antihyperglycemic agents					0.109
NO	1 (reference),1	1.79(0.49,6.52),0.379	2.05(0.61,6.9),0.246	1.64(0.44,6.07),0.46	
YES	1 (reference),1	1.09(0.32,3.66),0.89	2.47(0.89,6.91),0.084	4.86(1.66,14.27),0.004	
Use of insulin					0.19
NO	1 (reference),1	1.32(0.12,14.78),0.822	5.58(0.65,47.52),0.116	7.84(0.92,66.56),0.059	
YES	1 (reference),1	1.77(0.68,4.61),0.243	1.89(0.8,4.48),0.146	2.86(1.13,7.24),0.026	

*HR* Odds ratio, *CI* Confidence interval, *CKD* Chronic kidney disease, *SBP* Systolic blood pressure, *BMI* Body mass index, *RAASi* Renin–angiotensin–aldosterone system inhibitors

### Comparison of JRPS classification with RPS glomerular class and the total renal chronicity grade

**Fig. 4:**
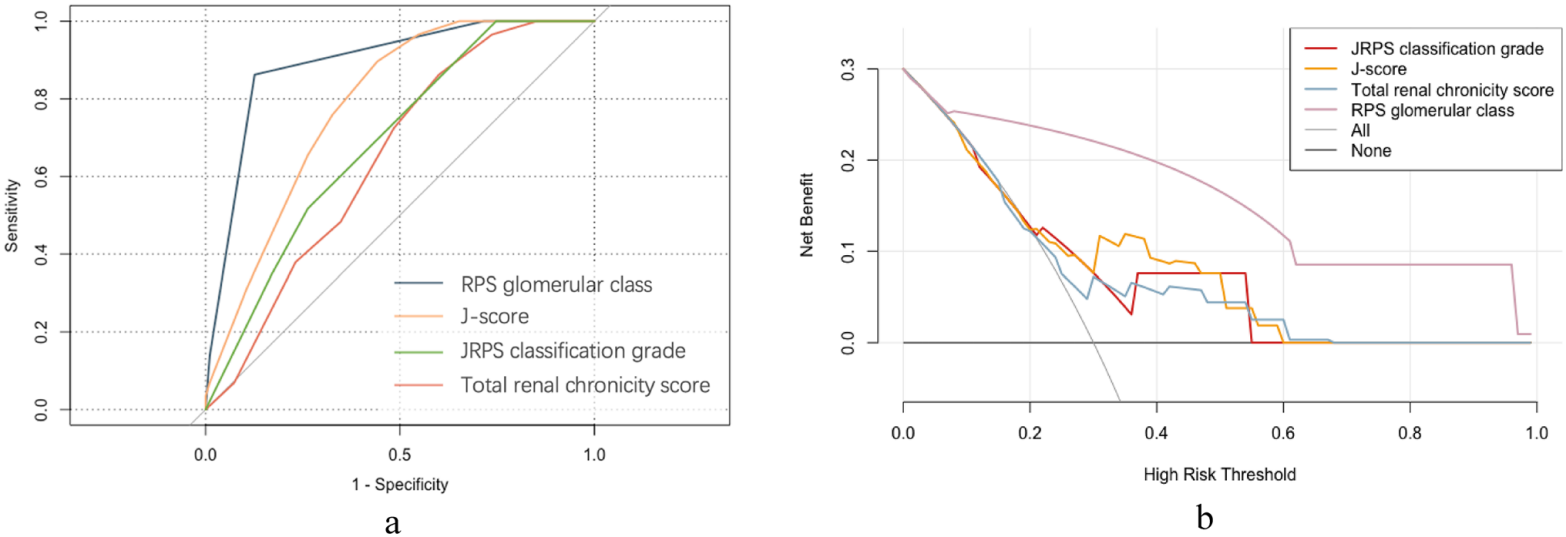
Comparison of **a** ROC curves and **b** decision curve analysis between different pathological models. *JRPS* Japanese renal pathology society, *J-score* JRPS score, *RPS* Renal pathology society

DeLong test showed that the RPS glomerular classification had significantly superior predictive performance compared to the other pathological scoring systems. The area under the ROC curve (AUC) for the RPS classification was 0.892, which was significantly higher than that for the total renal chronicity score (AUC: 0.892; 95% CI, 0.833–0.945, *P* < 0.0001), the JRPS classification grade (AUC: 0.690; 95% CI, 0.590–0.790, *P* < 0.0001), and the J-score (AUC: 0.784; 95% CI, 0.702–0.866, *P* = 0.018) (Fig. 4a).

Similarly, decision curve analysis (DCA) demonstrated that the J-score provided a higher net clinical benefit compared with the JRPS classification grade and the total renal chronicity score across a range of threshold probabilities (Fig. 4b). The details of the prediction performance are presented in Table 6.

**Table 6:** Comparison of predictive performance among pathological models for renal outcomes

	RPS glomerular class	J-score	JRPS classification grade	Total renal chronicity score
− 2 log-likelihood ratio	75.58	123.65	125.49	127.89
Hosmer–Lemeshow χ2 goodness-of-fit test	0.107	0.807	0.395	0.371
AUC (95% CI)	0.892(0.833–0.945)	0.784(0.702–0.866)	0.690(0.590–0.790)	0.648(0.547–0.750)
P vs RPS glomerular class (DeLong)	Reference	0.018	< 0.001	< 0.001
P vs J-score (DeLong)	0.018	Reference	0.059	0.024
NRI vs J-score	0.729(0.368–1.037)	Reference	− 0.454(− 0.839–0.051)	− 0.769(− 1.132–0.377)
IDI vs J-score	0.268(0.165–0.377)	Reference	− 0.097(− 0.165–0.033)	− 0.056(− 0.233–0.079)

*AUC* Area under curve, *CI* Confidence interval, *JRPS* Japanese renal pathology society, *J-score* JRPS score, *RPS* Renal pathology society, *NRI* Net reclassification improvement, *IDI* Integrated discrimination improvement

Reclassification analyses (NRI and IDI) further supported these findings. The RPS classification achieved the highest improvement in model discrimination (NRI = 0.729; IDI = 0.268) compared with the J-score, whereas the JRPS classification grade and total renal chronicity score yielded negative reclassification indices, reflecting reduced discriminative accuracy.

Furthermore, model calibration and overall goodness-of-fit were evaluated using the − 2 log-likelihood ratio and the Hosmer–Lemeshow χ^2^ goodness-of-fit test. As shown in Table 6, the J-score model demonstrated closer agreement between predicted and observed outcomes and achieved a better overall model fit compared with the JRPS classification grade model, further supporting its superior predictive performance in this cohort. (Table 6).

## Discussion

In this study, we retrospectively analyzed the clinical and pathological data of 124 patients with DKD and evaluated the prognostic utility of the JRPS classification in predicting renal outcomes. We observed that higher JRPS classification grades were associated with worse renal function parameters, including higher serum creatinine, more severe proteinuria, and an increased risk of composite renal outcomes. Correlation analysis further indicated that pathological features such as subendothelial space widening, exudative lesions, perihilar neovascularization, global glomerulosclerosis, and IFTA were positively associated with elevated serum creatinine and BUN levels.

Furthermore, multivariate Cox regression and subgroup analyses identified the JRPS classification grade as an independent prognostic factor for renal outcomes in patients with DKD. Even after adjustment for potential confounders—including age, sex, duration of diabetes, serum creatinine, and urinary protein—the JRPS classification grade remained significantly associated with an increased risk of adverse renal events. Notably, the association between higher JRPS classification grades and poor renal prognosis was more pronounced among elderly patients, males, and those with advanced CKD, indicating consistent prognostic value across diverse clinical subgroups. Collectively, these findings suggest that the JRPS classification grade may serve as a practical tool for clinicians to estimate individualized risk and monitor disease progression in DKD.

Additionally, we found a significant correlation between the JRPS classification grade and other established renal pathology scoring systems. Although JRPS classification grade provides a more comprehensive evaluation encompassing glomerular, tubulointerstitial, and vascular lesions, its predictive accuracy for renal outcomes (AUC = 0.690) was lower than that of the RPS glomerular classification (AUC = 0.892; DeLong *P* < 0.001). The J-score (AUC = 0.784) performed better than JRPS classification grade and the total renal chronicity score, but still lower than RPS classification. These results suggest that, while the JRPS classification grade and J-score systems capture broader histopathological changes and may complement traditional glomerular-based evaluation, the RPS glomerular classification still exhibits superior prognostic discrimination in DKD.

Previous studies have identified both interstitial disease and glomerular injury as independent predictors of progression to ESKD. [9, 10] In particular, an American study on renal biopsy in DKD demonstrated that interstitial fibrosis and renal tubular atrophy were significant predictors of clinical prognosis in univariate analysis. [11] These findings have been corroborated by other research, which highlighted the IFTA score and vascular disease score as key determinants of renal outcomes. [12] Therefore, a comprehensive pathological assessment of DKD should encompass not only glomerular lesions but also tubulointerstitial and vascular alterations to more accurately characterize the overall extent of renal damage.

Consistent with the findings of Taeyeong Kim, [5] our study demonstrated that the prognostic significance of the JRPS classification was lower than that of the RPS glomerular classification, which primarily emphasizes glomerular lesions. One possible explanation is that the J-score derived from the summation of multiple pathological parameters, may include factors that are not directly associated with renal prognosis, thereby attenuating its predictive precision. Although the JRPS classification provides a more comprehensive depiction of renal pathology—analogous to the ISN/RPS classification for lupus nephritis and the Oxford classification for IgA nephropathy [13, 14]—this broader inclusiveness did not translate into improved prognostic performance in our cohort. Therefore, the JRPS classification may be more appropriately considered as a complementary pathological framework that enhances contextual interpretation rather than a replacement for the RPS system in prognostic evaluation.

We performed an exploratory analysis of the association between the JRPS classification and cardiovascular outcomes. Diabetes mellitus markedly increases cardiovascular risk, especially in patients with diabetic nephropathy [15, 16]. The extent of renal dysfunction and albuminuria correlates closely with cardiovascular complications, and pathological changes such as mesangial expansion and arteriolar sclerosis have been linked to higher risks of mortality and major adverse cardiovascular events (MACE).

In our study, patients with higher JRPS classification grades showed a numerically higher incidence of cardiovascular outcomes, but this difference did not reach statistical significance, which is consistent with the findings reported by Furuichi et al. [17] Compared with the Japanese JRPS study, patients in our cohort were younger (52.1 vs. 57.8 years), had a lower proportion of males (41.2% vs. 67%), exhibited higher urinary protein levels (3.71–6.50 g/d vs. 1.77 g/d), and had lower RAASi usage (37.9% vs. 58.3%). These differences in baseline characteristics may have contributed to the lower cardiovascular event rates in our population. Moreover, the relatively shorter follow-up duration (36–56 months vs. 72 months) and potential residual confounding may have further limited our ability to detect significant associations.

To further investigate the relationship between JRPS classification grade and cardiovascular risk, we conducted a landmark analysis using 32 months as the cut-off point. This time point corresponded to the median follow-up duration among patients who reached renal outcomes, allowing for adequate evaluation of cardiovascular outcomes within a comparable observation window. Moreover, it represented the period during which most patients had completed essential follow-up visits, thereby maintaining sufficient statistical power. The landmark analysis revealed an increased incidence of cardiovascular events after 32 months, suggesting that higher JRPS classification grades may be associated with elevated long-term cardiovascular risk. The landmark analysis revealed a trend toward a higher incidence of cardiovascular outcomes beyond 32 months among patients with advanced JRPS classification grades, suggesting a possible association between more severe renal pathology and long-term cardiovascular risk. However, this association did not reach statistical significance; therefore, these findings should be interpreted with caution and considered hypothesis-generating.

Importantly, the JRPS classification system is designed to assess intrarenal structural lesions and does not capture systemic vascular pathology, such as endothelial dysfunction or atherosclerotic burden, which are key determinants of cardiovascular risk. This focus on localized renal injury may explain its weaker predictive performance for cardiovascular disease compared with biomarkers that reflect systemic vascular involvement.

Overall, our findings highlight the limitations of JRPS in evaluating cardiovascular outcomes and emphasize the need for future research involving larger and more diverse cohorts, longer follow-up durations, and integration of both renal and systemic markers to better define its role in cardiovascular risk prediction among patients with DKD.

The strengths of this study include the availability of a comprehensive pathological dataset and the first application of the JRPS classification in a domestic Chinese DKD cohort. Building upon the RPS classification proposed by Tervaert in 2010, the JRPS classification incorporates additional glomerular parameters [17] and extends the evaluation to tubulointerstitial and vascular lesions, providing a broader description of renal pathology.

Nevertheless, several limitations should be acknowledged. First, this was a single-center retrospective study with a relatively small sample size, which may limit the generalizability of our findings. Second, we did not formally assess interobserver reproducibility of pathological scoring, which is essential for validating the robustness of pathological classification. Third, because patients were enrolled up to 2020, data on newer therapeutic agents, such as SGLT2 inhibitors, were incomplete and might have influenced renal outcomes. Finally, our analysis primarily focused on pathological predictors, future studies should consider integrating JRPS classification with clinical biomarkers [18, 19] or emerging approaches such as artificial intelligence [20] to develop more comprehensive prognostic models. Genetic, lifestyle, and environmental factors may also contribute to DKD progression and warrant further investigation [21].

In conclusion, although the JRPS classification —particularly the J-score—provides a more comprehensive pathological evaluation, it demonstrates lower discriminatory power for renal prognosis compared to the RPS classification in this cohort. Nevertheless, the JRPS classification may offer complementary prognostic information and assist clinicians in assessing the risk of renal progression in patients with DKD. Further validation in larger, prospective cohorts is needed, but the JRPS classification holds potential to enhance prognostic assessment and guide individualized clinical decision-making in DKD.

## Supplementary Information

Below is the link to the electronic supplementary material.

Supplementary file1 (DOCX 452 KB)
