# Supplementary material for: Prognostic assessment of the Japanese Renal Pathology Society classification in Chinese patients with histologically confirmed diabetic kidney disease
Source: Clin Exp Nephrol. 2025 Nov 26;30(2):275–85. doi: 10.1007/s10157-025-02782-w (PMC12886328; doi:10.1007/s10157-025-02782-w)
Supplement: Supplementary file 1 — Supplementary file1 (DOCX 452 KB) [file 10157_2025_2782_MOESM1_ESM.docx]

### Supplementary Table S1. Univariate cumulative odds COX regression analysis in DKD patients

| Variables | Univariate Cox Regression | |
| --- | --- | --- |
|  | HR (95%CI) | P-value |
| Hemoglobin(g/L) | 0.98 (0.97, 0.99) | <0.001 |
| HbA1c（%） | 0.95 (0.85,1.07) | 0.406 |
| FBG (mmol/L) | 1.01 (0.97,1.05) | 0.710 |
| Scr (μmol/L) | 1.01 (1.01,1.01) | <0.001 |
| BUN (mmol/L) | 1.06 (1.03,1.09) | <0.001 |
| Serum albumin (g/L) | 0.92 (0.90, 0.95) | <0.001 |
| eGFR (ml/min/1.73 m2) | 0.98 (0.98, 0.99) | <0.001 |
| Urinary protein (g/d) | 1.10 (1.07,1.13) | <0.001 |
| JRPS classification grade |  |  |
| 1 | 1.00 (Reference) |  |
| 2 | 1.51 (0.64,3.57) | 0.350 |
| 3 | 2.36 (1.08,5.15) | 0.031 |
| 4 | 3.33 (1.46,7.59) | 0.004 |
| J-score | 1.12 (1.06,1.18) | <0.001 |
| Diffuse（0/1/2/3） |  |  |
| 0 | --- | --- |
| 1 |  | 1 (reference) |
| 2 | 1.36(0.87,2.11) | 0.177 |
| 3 | 2.03(1.08,3.83) | 0.029 |
| Nodular（0/1） | 3.97(2.44,6.46） | <0.001 |
| SubendW（0/1/2/3） |  |  |
| 0 | --- | --- |
| 1 |  | 1 (reference) |
| 2 | 7.34(3.68,14.64) | 0.003 |
| 3 | 10.33(4.99,21.39) | <0.001 |
| Exudative (0/1) | 1.93(1.28,2.91) | 0.002 |
| MesLy (0/1) | 1.96(1.25,3.07) | 0.003 |
| PVas (0/1) | 2.32(1.52,3.54) | <0.001 |
| GScl (%) | 1.00(0.99,1.02) | 0.113 |
| SScl (%) | 1.00(0.99,1.02) | 0.584 |
| GMeg (0/1) | 1.68(1.08,2.60) | 0.02 |
| IFTA (0/1/2/3) |  |  |
| 0 |  | 1 (reference) |
| 1 | 1.43(0.41,4.92) | 0.573 |
| 2 | 2.35(0.68,8.20) | 0.179 |
| 3 | 3.53(1.03,12.16) | 0.045 |
| ICell (0/1/2) |  |  |
| 0 |  | 1 (reference) |
| 1 | 0.57(0.18,1.84) | 0.346 |
| 2 | 0.92 (0.29,2.95) | 0.886 |
| Hyalin (0/1/2/3) |  |  |
| 0 |  | 1 (reference) |
| 1 | 1.22(0.79,1.88) | 0.380 |
| 2 | --- | --- |
| 3 | 1.20(0.62,2.30) | 0.586 |
| IntThic (0/1/2) |  |  |
| 0 |  | 1 (reference) |
| 1 | 0.82(0.20,3.33) | 0.776 |
| 2 | 0.91(0.25,4.82) | 0.914 |

### JRPS, Japanese Renal Pathology Society; Diffuse diffuse lesion (mesangial expansion); Nodular nodular lesion (nodular sclerosis), SubendW subendothelial space widening (double contour of basement membrane); Exudative exudative lesion; MesLy mesangiolysis/microaneurysm; PVas perihilar neovascularization (polar vasculosis); GScl global glomerulosclerosis/collapsing glomerular change ischemic glomerular change; SScl segmental glomerulosclerosis; GMeg glomerulomegaly; IFTA interstitial fibrosis and tubular atrophy; ICell interstitial cell infiltration; Hyalin arteriolar hyalinosis; Arterio Arteriosclerosis with intimal thickening; Scr serum creatinine, eGFR estimated glomerular filtration rate; BUN blood urea nitrogen; FBG fasting blood glucose

### Supplementary Table S2 Effect of J-score on renal outcomes.

| Variables | Model1 | |  | Model2 | |  | Model3 | |
| --- | --- | --- | --- | --- | --- | --- | --- | --- |
|  | HR (95%CI) | *P* |  | HR (95%CI) | *P* |  | HR (95%CI) | *P* |
| J-score | 1.12(1.06,1.18) | <0.001 |  | 1.12 (1.05,1.18) | <.001 |  | 1.11(1.05,1.18) | <0.001 |

### Supplementary Table S3 Effect of RPS glomerular class on renal outcomes.

| Variables | Model1 | |  | Model2 | |  | Model3 | |
| --- | --- | --- | --- | --- | --- | --- | --- | --- |
|  | HR (95%CI) | *P* |  | HR (95%CI) | *P* |  | HR (95%CI) | *P* |
| RPS glomerular class |  |  |  |  |  |  |  |  |
| Grade 1,2 | 1.00 (Reference) |  |  | 1.00 (Reference) |  |  | 1.00 (Reference) |  |
| Grade 3,4 | 7.27(3.73,14.17) | <0.001 |  | 7.42 (3.79,14.52) | <0.001 |  | 6.03 (3.03,12.01) | <0.001 |

Supplementary Table S4 Effect of Total renal chronicity grade on renal outcomes.

| Variables | Model1 | |  | Model2 | |  | Model3 | |
| --- | --- | --- | --- | --- | --- | --- | --- | --- |
|  | HR (95%CI) | *P* |  | HR (95%CI) | *P* |  | HR (95%CI) | *P* |
| Total renal chronicity grade |  |  |  |  |  |  |  |  |
| Grade 1,2 | 1.00 (Reference) |  |  | 1.00 (Reference) |  |  | 1.00 (Reference) |  |
| Grade 3,4 | 1.89(1.25,2.88） | 0.003 |  | 1.84 (1.21,2.80) | 0.004 |  | 1.42 (0.83, 2.40) | 0.199 |

Model1: Crude

Model2: Adjust: age, gender

Model3: Adjust: age, gender, Urinary protein, Duration of Diabetes, Scr, use of RAASi, statins, CCB

HR: Hazard Ratio; CI: Confidence Interval; JRPS, Japanese Renal Pathology Society; Scr serum creatinine; RAASi renin-angiotensin-aldosterone system inhibitors; CCB calcium-channel blocker

Supplementary Figure S1. Flowchart of study participants.


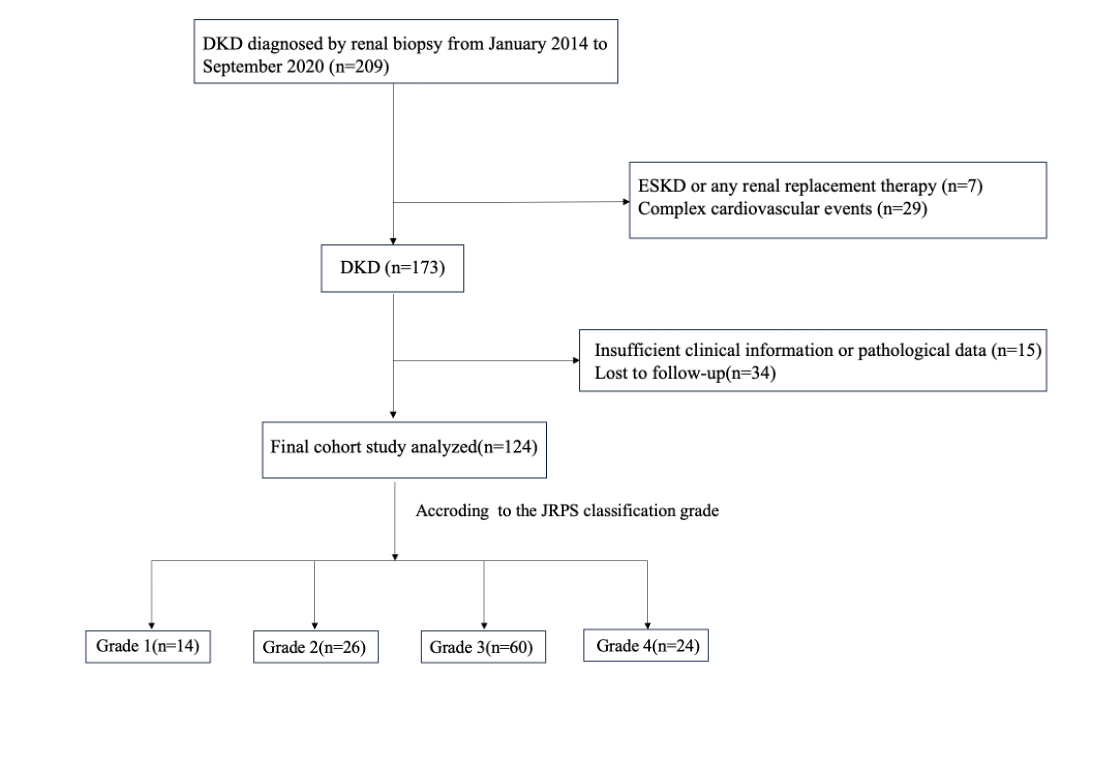


- DKD, diabetic kidney disease; ESKD: end-stage kidney disease.

Supplementary Figure S2. Representative boxplots with overlaid dot plots showing the associations between pathological grades and clinical variables.A. Correlation analysis between eGFR and histopathological parameters; B. Correlation analysis between scr and histopathological parameters; C. correlation analysis between urinary protein and histopathological parameters.


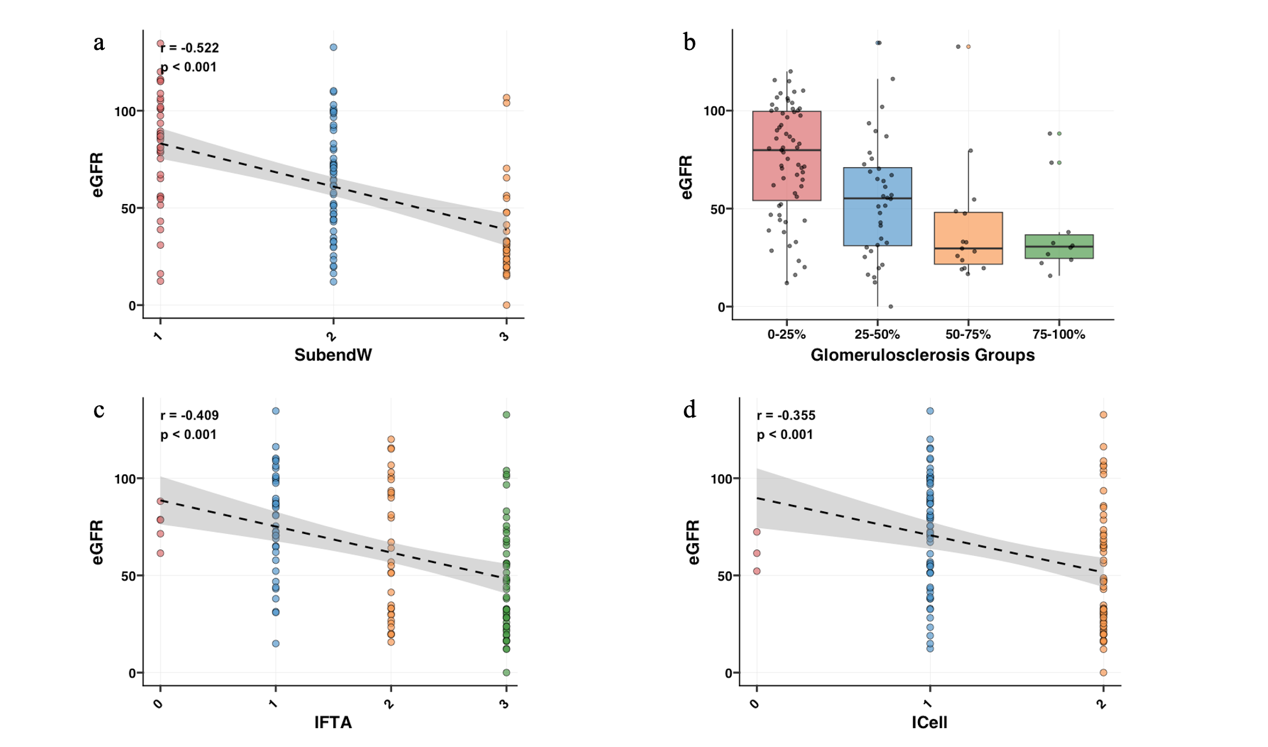


A


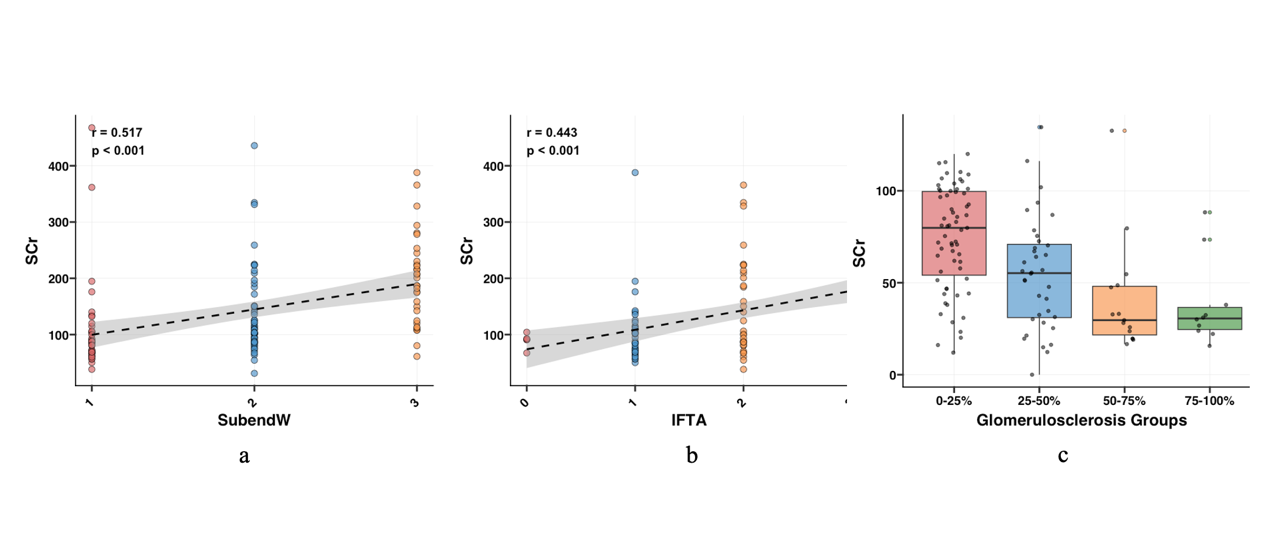


B


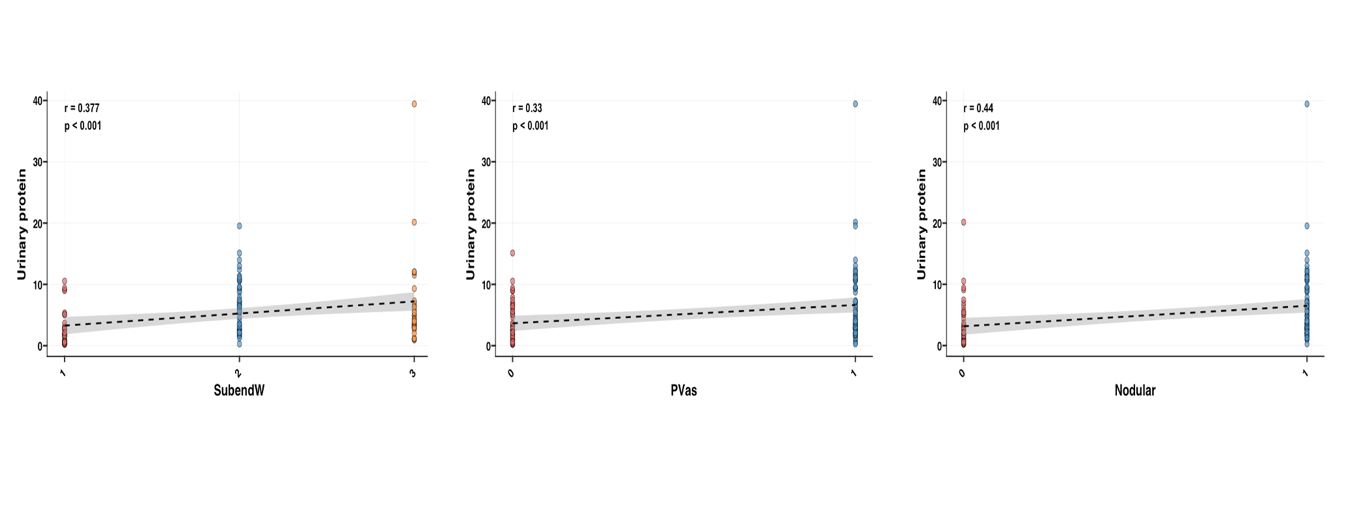


C

### Nodular nodular lesion (nodular sclerosis), SubendW subendothelial space widening (double contour of basement membrane); PVas perihilar neovascularization (polar vasculosis); GScl global glomerulosclerosis/collapsing glomerular change ischemic glomerular change; IFTA interstitial fibrosis and tubular atrophy; ICell interstitial cell infiltration; eGFR estimated glomerular filtration rate; Scr serum creatinine
